# Graves’ disease as a driver of depression: a mechanistic insight

**DOI:** 10.3389/fendo.2023.1162445

**Published:** 2023-04-20

**Authors:** Yifei Song, Xinying Wang, Wenxin Ma, Yan Yang, Shuxin Yan, Jiapan Sun, Xiaoyun Zhu, Yang Tang

**Affiliations:** ^1^Beijing University of Chinese Medicine, Beijing, China; ^2^Centre for Evidence-Based Chinese Medicine, Beijing University of Chinese Medicine, Beijing, China; ^3^Tongling Municipal hospital, Anhui, China; ^4^Department of Geriatrics, Peking University Shenzhen Hospital, Shenzhen Peking University-The Hong Kong University of Science and Technology Medical Center, Shenzhen, Guangdong, China; ^5^Guang’anmen Hospital, China Academy of Chinese Medical Sciences, Beijing, China

**Keywords:** Graves’ disease, depression, autoimmune, gut, hormone

## Abstract

Graves’ disease (GD) is characterized by diffuse enlargement and overactivity of the thyroid gland, which may be accompanied by other physical symptoms. Among them, depression can dramatically damage patients’ quality of life, yet its prevalence in GD has not received adequate attention. Some studies have established a strong correlation between GD and increased risk of depression, though the data from current study remains limited. The summary of mechanistic insights regarding GD and depression has underpinned possible pathways by which GD contributes to depression. In this review, we first summarized the clinical evidence that supported the increased prevalence of depression by GD. We then concentrated on the mechanistic findings related to the acceleration of depression in the context of GD, as mounting evidence has indicated that GD promotes the development of depression through various mechanisms, including triggering autoimmune responses, inducing hormonal disorders, and influencing the thyroid-gut-microbiome-brain axis. Finally, we briefly presented potential therapeutic approaches to decreasing the risk of depression among patients with GD.

## 1 Introduction

Graves’ disease (GD) is an autoimmune condition of the thyroid caused by the excessive production of stimulatory antibodies (thyroid-stimulating antibodies, TSAb) against the thyroid-stimulating- hormone receptor (TSH-R). It is the most common cause of hyperthyroidism (1). Epidemiological studies indicate that the incidence of GD is 20–40 cases per 100,000 population per year (2). Research has associated hyperthyroidism with severe work disability (3), and the development of various diseases across different systems in the onset of GD has been well documented (4–6). These complications dramatically worsen the clinical outcomes of GD patients and are associated with increased mortality (7) and economic burden. With recent advances in neuroendocrinology, psychiatric disorders in GD patients are of growing interest to researchers.

Previous research has related overt hyperthyroidism with psychiatric disorders such as irritability, anxiety, mania, sleeplessness, and depression (8, 9). In recent years, increasing evidence has indicated the high prevalence of depression and related symptoms in patients with GD (10–12). As GD hyperthyroidism is more commonly manifested as emotional agitation and irritability (4, 13) and depression is usually linked with hypothyroidism instead of hyperthyroidism, the mechanism behind GD and depression seems far less clear compared with other moods disorders observed in GD. Studies have predicted that the development of depression in GD may be related to the exhaustion of noradrenergic transmission (14). Yet, this assumption has only considered the traditional theory of the cause of hyperthyroidism, meaning that more complicated mechanisms involving autoimmunity, hormone metabolism, gut dysbiosis, etc., may be ignored.

As a result of the inability to understand the mechanism of depression and related symptoms in GD, depression in GD may well be overlooked, which can lead to severe clinical consequences. Characterized by a persistent feeling of sadness and/or an inability to experience pleasure, depression is widely linked to serious consequences. The World Health Organization has ranked it the second most significant cause of disability worldwide (15–17). Thus, the development of depression will not only seriously impair the quality of life of GD patients but also aggravate the original condition of GD and affect its prognosis. In spite of this, depression, along with other mood disorders, has not been listed in GD guidelines as an intervention target (18). These realities call for more attention in understanding GD and depression, which we hope will provide a more systematic vision of the crosstalk between thyroid functions and the neuropsychiatric system, as well as benefiting from the improvement of treatment methods of GD and lowering its recurrence rate.

This article aims to provide mechanistic insights into depression in GD. Through summarizing current literature in this field, the driving role of GD in the development of depression has been revealed. In this article, we will first review epidemiological evidence supporting the increased risk of depression among GD patients. We will then highlight the pathogenesis links between GD and depression from three different aspects. Finally, we will briefly present potential preventative and treatment methods for depression in the context of GD. The main objective of this article is to outline the mechanism of depression in the context of GD, so as to provide references for future studies on the prevention and treatment of GD emotional complications.

## 2 Graves’ disease and increased risk of depression

GD is a prevalent autoimmune thyroid syndrome that has been linked to a wide range of illnesses, including cardiovascular diseases (19), hepatic dysfunction (20), neurological diseases (6), and so on. Among them, mental disorders, mainly depression and anxiety, are found to be closely related to GD (21).

Previous and updated epidemiological studies have uncovered the relationship between GD and depression. To review the bodies of evidence, we systematically screened on PubMed/MEDLINE, Cochrane Library, and Web of Science from the database inception to March 22, 2023. We combined generic terms for GD, hyperthyroidism, depression, and population-based study designs. After the exclusion of duplicates, 11 studies were screened, which included 5 cohort studies, 3 cross-sectional studies, and 3 case-control studies (**Table 1**).

**Table 1:** Clinical studies of Graves' disease related to depression.

References	Publication Year	Country	Study Population	Study Design	Length of Follow-up	Main findings
Chen HH et al	2014	China	20975	Retrospective cohort	11	GD patients were associated with significantly higher risk of depression (HR 1.69(1.45-1.96))
Ittermann T et al	2015	Germany	6267	Cross-sectional	5	Diagnosed untreated hyperthyroidism is associated with depression.
Kvetny J et al	2015	Denmark	23001	Cross-sectional	3	Subclinical hyperthyriroidism seem to have a risk, although small, of subclinical depression.
Hong JW et al	2018	Korea	1763	Cross-sectional	1	Subclinical hyperthyroidism was independently associated with depressive symptoms in the Korean general population using national cross-sectional data.
Fetene DM et al	2020	Britain	5840	Cohort	2	Increased levels of FT4 during the first trimester of pregnancy appear be linked to greater risk of offspring depression.
Yuan L et al	2020	China	127	Cohort	_	Patients with thyroid dysfunction (31 hypothyroid, 32 subclinical hypothyroidism, 34 hyperthyroid, and 30 subclinical hyperthyroidism) had various degrees of anxiety and depression disorders.
Pápai A et al	2021	Hungary	31	Case control	_	Both patients with hyperthyroidism and with hypothyroidism had high levels of depression and anxiety.
Hamed SA et al	2021	Egypt	75	Case control	_	GD is associated with higher frequencies and severities of anxiety, depression and inattention during periods of thyroid hormone elevation.
Yang L et al	2021	China	1803	Retrospective cohort	13	High serum FT3 levels and comorbidity of thyroid disease could increase the risk of readmission after hospitalization for MDD.
Leone M et al	2022	Sweden	2200000	Cohort	40	Individuals with endocrine-metabolic disorders including Graves' disease had a significantly higher risk of depression.
Stanić G et al	2022	Serbia	335	Case control	_	Psychosomatic symptoms were significantly more severe in patients with thyroid disorders compared to the control group.

Three studies, including 2 cohort studies and 1 case-control study, have pointed out the direct correlation between GD and increased risk or severities of depression. Chen et al. conducted a retrospective cohort study involving 20975 patients (4195 GD patients and 16780 non-GD patients) and discovered that GD and GD treatment are associated with an increased risk of depression in Asian patients (22). Another nationwide Swedish cohort study has also pointed out a significantly higher risk of depression in GD (23). Moreover, as GD is the most common cause of hyperthyroidism (4), studies on hyperthyroidism have also provided evidence for the correlation between GD and depression. A cross-sectional study of 2142 individuals has found an association between untreated hyperthyroidism and a higher risk of depression (24). Another Korean national cross-sectional data study concluded that subclinical hyperthyroidism was independently associated with depressive symptoms. Additionally, studies by Pápai A et al. (25) and Hamed SA et al. (12) have supported the correlation between hyperthyroidism and depression.

Although our screening results have already supported a strong link between GD and depression, the number of current clinical studies available remains very limited. Further prospective longitudinal studies will be needed to determine whether GD affects the pathophysiology of depression.

## 3 Graves' disease as a mechanistic driver of depression

**Figure 1:**
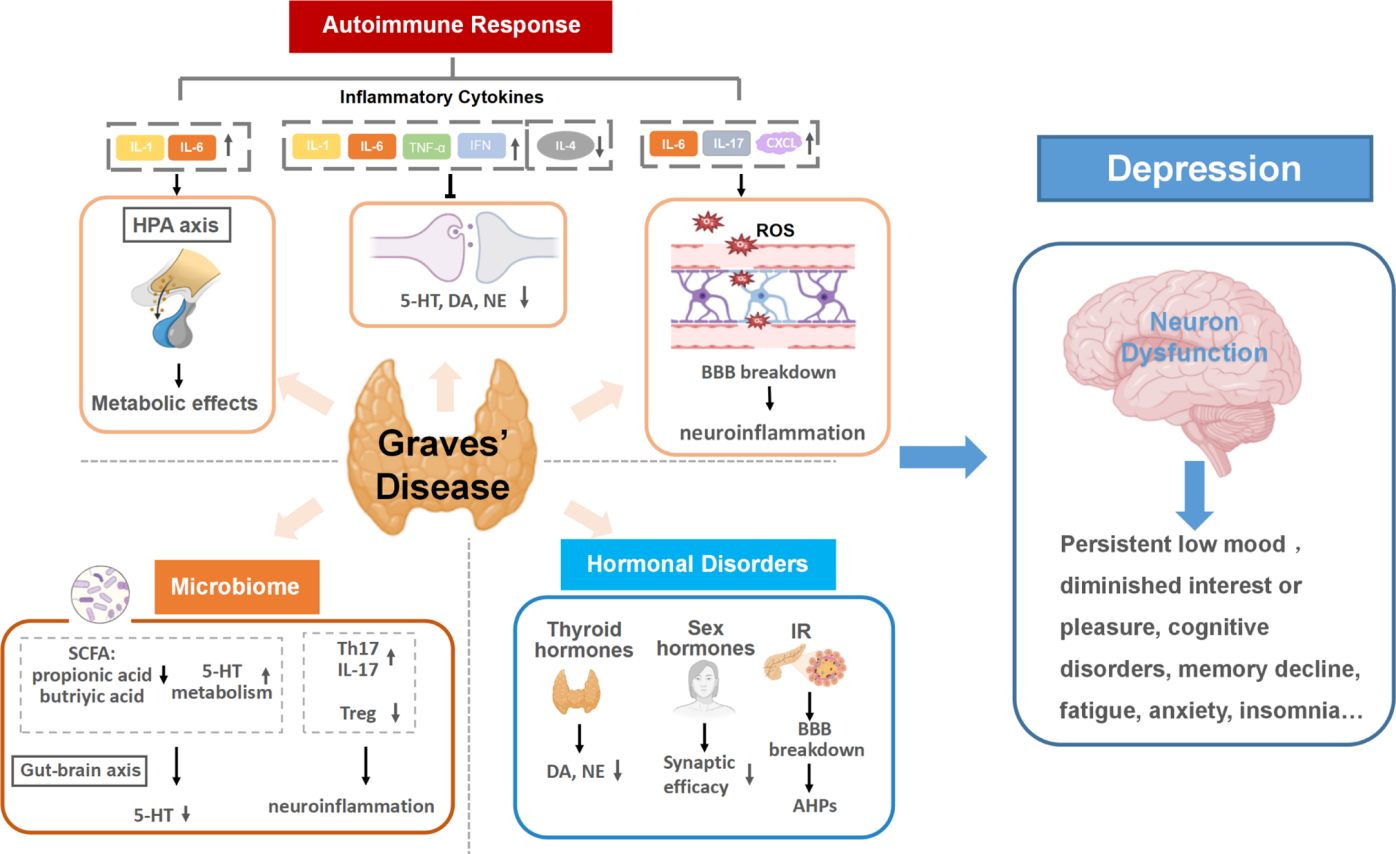
Graves’ disease as a mechanistic driver of depression. Specific pathways of autoimmune responses, hormonal disorders and microbiota dysbiosis in GD trigger neuron dysfunction in the brain and promote the development of depression. (1) Autoimmune responses in GD cause altered inflammatory cytokines levels. Continuous activation of the HPA axis induced by persistent inflammatory cytokines stimulation can damage neurons and activate continuing central inflammation, along with affecting the expression and action of hormone receptors. Inflammatory cytokines can also disrupt monoamine neurotransmitter metabolism, resulting in lower accessibility to these neurotransmitters. The increase of BBB permeability in the inflammatory state of GD allows inflammatory cytokines to enter the brain and may lead to neuroinflammation. (2) Multiple hormonal disorders can develop as a result of GD hyperthyroidism, including thyroid hormones, insulin and sex hormones. The increase of thyroid hormones can reduce the secretions of dopamine (DA) and norepinephrine (NE). Insulin resistance (IR) in GD can increase both blood and intracellular glucose level, inducing neuronal tissue damage and afterhyperpolarization (AHP). Additionally, sex hormone disorders in GD are likely to lower Synaptic efficacy, which is related to the pathogenesis of depression. (3) Gut dysbiosis in GD can result in lower accessibility to 5-HT and activate host immune responses, increasing the risk of depression. HPA axis, the hypothalamic-pituitary-adrenal axis; 5-HT, serotonin; IR, insulin resistance; DA, dopamine; NE, norepinephrine; BBB, the blood-brain barrier; SCFA, short-chain fatty acids; AHP, afterhyperpolarization; 5-HT, 5-hydroxytryptamine.

Mounting evidence has indicated that GD promotes the development of depression through a variety of mechanisms, including triggering autoimmune responses, inducing hormonal disorders, and influencing the thyroid-gut-microbiome-brain axis. (Shown in **Figure 1**).

### 3.1 Autoimmune and inflammatory responses in GD increase the risk of depression

GD is a common autoimmune disease in which excessive production of stimulatory autoantibodies disrupts critical physiological processes inside and outside the thyroid gland in GD, resulting in cellular and humoral immune disorders (26). Earlier studies have demonstrated the relationship between depression and immune disorders, with a focus on the role of inflammation and proinflammatory cytokines (27). A more recent study by Fam et al. observed significantly higher TSH receptor antibodies (TRAbs) concentration in depressed patients than in healthy controls, which suggested that autoimmunity responses in GD may exert neuropsychiatric effects, which increase susceptibility to depression (28).

Cytokines play an important role in autoimmune responses and are often used as indicators to predict the occurrence and progression of diseases (29). Previous studies have shown that cytokines can enter and influence the central nervous system through nerves, body fluids, the blood-brain barrier(BBB), cytokine receptors, and other pathways (30). As indispensable components of immunity, lymphocytes, and their specific cytokines are shown to play a pathogenic role during the course of GD by previous studies (31). These cytokines can lead to various subsequent neuropsychiatric effects, including disrupting neurotransmitter metabolism, inducing BBB dysfunction, and dysfunction of the hypothalamic-pituitary-adrenal (HPA) axis, playing a vital role in the development of depression.

#### 3.1.1 Disrupting neurotransmitter metabolism

Neurotransmitters are vital chemicals that transmit information between neurons or neurons and effectors. Studies have shown that depression may involve abnormalities of numerous transmitter systems and molecular mechanisms across specific neural brain structures. For example, the mesolimbic dopaminergic pathway composed of dopaminergic (DA) neurons in the ventral tegmental area (VTA) and their projections to the nucleus accumbent (NAc) is crucial for the recognition of emotionally salient stimuli such as reward and aversion. Decreased DA neuron activity in VTA is associated with depression-related behaviors (32). As cytokines can regulate the metabolism and availability of neurotransmitters, they may play a driving role in the above pathological processes.

Changes in levels of various cytokines, which have potential effects on neurotransmitter metabolism, have been widely observed in the thyroid and orbital tissues of GD patients (33). These changes can be mainly summarized as increased pro-inflammatory cytokines and decreased anti-inflammatory cytokines. Ujhely et al. compared the levels of proinflammatory cytokines in tears between GD patients and healthy controls and discovered that the release of IL-1β, IL-6, IL-13, IL-17A, IL-18, TNF-α, and RANTES significantly increased in patients with GD and Graves ophthalmopathy (GO) (34). Elevated concentration of circulating IL-6/sIL-6R was also discovered in patients with GD (35). Additionally, Ruiz-Riol et al. (36) found overexpression of interferon (IFN) signal after bioinformatics analysis of the thyroid gland in GD. By contrast, a study by Kallmann et al. discovered that the release of IL-4, a typical anti-inflammatory cytokine, decreased in GD (37).

Subsequently, the alteration of these cytokines is involved in the disruption of monoamine neurotransmitters, including serotonin (5-HT), dopamine (DA), and norepinephrine (NE) (38, 39). In the metabolism processes of 5-HT, Indoleamine 2-dioxygenase (IDO) is a critical enzyme that catabolizes L-tryptophan (TRP) into L-kynurenine (KYN) by cytokine-induced activation. KYN is then converted into quinolinic acid (QUIN) and kynurenic acid (KA), which are neuroactive and may contribute to the behavioral changes experienced by some patients during exposure to inflammatory stimuli (40). Pro-inflammatory cytokines represented by IL-1, IL-6, TNF- α, and IFN can activate IDO, while anti-inflammatory cytokines such as IL-4 can down-regulate the enzyme. With increased pro-inflammatory cytokines and decreased anti-inflammatory cytokines in GD, IDO can be activated, resulting in greater tryptophan consumption and decreased 5-HT accessibility (41, 42). In addition to interfering with 5-HT metabolism, proinflammatory cytokines have also been reported to disrupt the synthesis, release, and reuptake of DA. By suppressing striatal DA release, INF-α may contribute to multiple depressive symptoms (43–45). TNF-α can induce delayed and progressive loss of DA neurons in the substantia nigra (SN) (46). Moreover, central inflammatory signals can activate mitogen-activated protein kinase (MAPK), which in turn increases the number and activity of presynaptic reuptake pumps, resulting in reduced synaptic availability of monoamine neurotransmitters, including norepinephrine (NE) (47, 48).

As neurotransmitters play a critical role in the function of the central nervous system, the disrupted metabolism of monoamine neurotransmitters can increase the risk of depressive behaviors. On the one hand, disruption of these neurotransmitter metabolisms can result in the loss of innervation of monoamine neurons in the brain and induces depressive behaviors such as mood disorders (45, 49, 50). On the other hand, inflammation, as previously indicated, triggers an increase in QUIN, which binds to the N-methyl-D-aspartate receptor (NMDAR), inducing the release of glutamate (48, 51). As an excitatory neurotransmitter, high level of glutamate can alter the strength of certain groups of glutamatergic synapses in a variety of brain regions, such as the prefrontal cortex (PFC), hippocampus, and nucleus accumbens (NAc), causing dysfunction of cortico-mesolimbic reward circuitry that underlies many manifestations of depression (52).

#### 3.1.2 Triggering neuroinflammation following BBB disruption

Neuroinflammation was mediated by the activation of microglial cells (53), the primary resident immune cells in the central nervous system, which in turn activates nuclear factor-kappaB (NF-κB) and produces inflammatory cytokines (54, 55). Its vital role in depression is increasingly recognized (56). Converging lines of evidence indicated that activation of immuno-inflammatory pathways is highly associated with developing various neuropsychiatric diseases (57). Studies also found that persistent low-grade inflammatory activation is related to the severity of depressive symptoms and the probability of treatment response (57).

The blood-brain barrier (BBB) is formed by endothelial cells sealed by tight junction proteins, pericytes, and astrocytes. The specific multicellular structure of BBB controls its major functions, which include maintaining brain homeostasis, regulating influx and efflux transport, and providing protection from injury (58). Various factors in GD can increase BBB’s permeability, which allows multiple inflammatory cytokines to enter the brain and contributes to the development of neuroinflammation. For instance, IL-17 and IL-6, which are typical cytokines observed in GD patients and animal models (35, 59), can disrupt the normal structure and function of the BBB. The effect of IL-17 on BBB disruption has been thoroughly documented (35, 59). Huppert et al. found that IL-17 increased reactive oxygen species (ROS) production and activated oxidative stress, activating endothelial contractile machinery. Activation of the contractile apparatus is responsible for the loss and disorganization of dense tight junction (TJ) proteins, which consecutively lead to BBB breakdown (60). Apart from IL-17, researchers also found that circulating IL-6 could act on the main cell adhesion component of endothelial cells, tight junction protein claudin5 (Cldn5), which alters BBB permeability (61). In addition to these two cytokines, lymphocytes in the thyroid secrete diverse pathogenic cytokines into the blood, resulting in circulating cytokine disorders and increased BBB’s permeability.

As a result of BBB disruption, inflammatory factors are more prone to entering the central nervous system and subsequently activate neuroinflammation. One typical pathway is through activating microglia. Microglia are inherent immune effector cells in the central nervous system, which play an essential role in regulating neuronal development, neuron apoptosis, and other physiological processes (62). A previous experiment showed that the up-regulation of IL-17 induced the activation of microglia in the hippocampus, amygdala, and prefrontal cortex ofmodel mice, resulting in depression-like behaviors in early adulthood than those in single or dual stress groups (63). Activated and polarized microglia typically exhibit the pro-inflammatory M1 phenotype and produce pro-inflammatory cytokines, contributing to neuronal dysfunction and reinforcing disease-related behaviors, all of which are related to psychiatric disorders (64, 65).

Another pathway is through recruiting immune cells by chemokines. Antonelli et al. observed elevated chemokine levels in patients with GD (66). C-X-C motif chemokine ligand (CXCL), a typical chemokine, can recruit immune cells to infiltrate the cerebrovascular system and brain parenchyma before initiating a variety of pathways leading to neuronal death (67). In addition, Th17 cells recruited by IL-17 can induce neuronal death by directly interacting with neurons or inducing elevated Ca^2+^ levels (68). Neuronal death thus causes the reduction of brain volume and hinders neuro-progression, which in turn contributes to depression. The atrophy of brain regions such as the hippocampus and prefrontal cortex was observed in patients with depression (69), which may be the result of decreased neuron number as a result of neuronal death. Research has also shown that neuronal death, neurodegeneration, decreased neurogenesis, and reduced neuroplasticity can jointly contribute to impaired neuro-progression (70). Impaired neuro-progression, which results in somatic symptoms including anhedonia, anxious behavior, fatigue, discomfort, and psychiatric symptoms such as mild cognitive impairment (MCI), is associated with the progression of depression (71, 72).

In summary, abnormal levels of GD-related inflammatory factors can disrupt the BBB before mediating the formation of neuroinflammation through multiple pathways, such as activation of central microglia and recruitment of immune cells, which can cause nerve damage and increase susceptibility to depression.

#### 3.1.3 Dysregulation of the HPA axis

The HPA axis is one of the critical physiological systems that regulate systemic inflammatory response and immune response (73). It secretes three main classes of hormones, which are regulated hierarchically. Sensory signals control the release of hypothalamic corticotropin-releasing hormone (CRH) and vasopressin (AVP) from the paraventricular nucleus and into the pituitary portal circulation, where they reach the adenohypophysis and stimulate the release of adrenocorticotropic hormone (ACTH), which in turn promotes adrenocortical production and cortisol release (74). From an evolutionary perspective, the activation of the ACTH/HPA axis and sympathetic spinal system, along with the diminished function of the prefrontal cortex, results in a diminished ability to seek and experience pleasure, reduced food intake, decreased sexual activity, and sleep apnea. These are transient adaptive changes the body makes in response to stress (75).

In the pathological state, due to the persistent stimulation of the central cytokine system, the CRH/HPA axis and the noradrenergic system will be continuously activated. IL-1 and IL-6, two proinflammatory cytokines elevated in GD (76–78), are typical cytokines involved in stimulating CRH/HPA axis hyperactivity. After binding to the IL-1 type I receptor, IL-1β could mediate the activation of the HPA axis in the rodent model of depression (79), which provides evidence for IL-1’s role in activating the HPA axis. As IL-1 has known effects on hypothalamic biogenic amines, the main source of corticotropin-releasing factor (CRF), it can regulate on pituitary ACTH and promote the production and release of cortisol (80), which may be the underlying mechanism of the HPA axis activation by IL-1. Apart from IL-1, the role of IL-6 in activating the HPA axis has also been found (81, 82).

The HPA axis is involved in the regulation of many physiological functions, such as energy balance, sleep-wake transition, and hippocampal neuron survival (83). Therefore, the disruption of the HPA axis is the pathological basis of many diseases. HPA axis abnormalities are related to the pathophysiology of cognitive impairment (84). Study data show that more than half of depressed patients have other HPA axis disorders, such as hypercortisolemia or altered cortisol circadian rhythm (85). The activated HPA axis is involved in the pathogenesis of depression by damaging neurons and continuing central inflammation. Adrenocortical steroids enter the brain and have widespread effects, particularly impairing hippocampal function (86). Long-term exposure to the hormone environment can decrease the secretion of derived neurotrophic factor (BDNF) and interfere with neural development (87, 88). Adrenocortical hormones not only destroy the plasticity and structure of neurons, but also exert cytotoxic effects by regulating neurotransmitters.

In addition to mediating HPA axis activation resulting in abnormal hormone secretion, cytokines also affect the expression and action of hormone receptors. The number of adrenergic receptors expressed in IL-1Ra gene knockout (IL-1RA KO) mice showed complex stage changes (89). The levels of IL-6, IL-10, and TNF-α in patients with depression can also affect the sensitivity of glucocorticoid receptors.

It has also been found that there is an interaction between the two major stress response systems in depression—The hypothalamic-pituitary-adrenal axis and the immune system (90). Glucocorticoids secreted by the HPA axis are biphasic immunomodulatory substances that balance anti-inflammatory and pro-inflammatory (91). In the absence of inflammation, basal levels of glucocorticoid receptor signaling promote the expression of PRRs, cytokine receptors, and complement factors, sensitizing cells to noxious stimuli and promoting the induction of inflammatory responses after tissue injury. However, in the inflammatory state, glucocorticoid concentrations suppress the immune response mainly by inhibiting the transmission of PRR, Fc receptors, and cytokine signaling, thereby shortening the duration of the immune response (92, 93). In depressed patients, the expression and availability of glucocorticoid receptors are decreased, which prevents the HPA axis from exerting anti-inflammatory effects and promotes persistent inflammation (94, 95). The increased activity of pro-inflammatory transcription factors and NF-κB has also been associated with decreased glutamate receptor activity (96).

Therefore, as an autoimmune disease, GD can cause systemic immune activation and high levels of inflammatory factors in the circulation, leading to the activation of the HPA axis and hypersecretion of related hormones. Continuous activation of the HPA axis weakens its anti-inflammatory functions, causing a persistent state in the body and increasing the risk of depression.

### 3.2 Multiple hormonal disorders in GD may increase the risk of depression

GD is not only an autoimmune disease but also an endocrine disease characterized by excessive secretion of thyroid hormones. The extracellular portion of TSHR on the surface of thyroid cells is recognized by GD autoantibodies - TRAbs, which causes hyperthyroidism (97). Subsequent abnormalities of various endocrine hormones are developed as a result of hyperthyroidism. Studies have confirmed that thyroid function will affect HPA axis activity, insulin sensitivity of glucose, lipid metabolism, and ovarian secretion function, resulting in hypercortisolism, insulin resistance, and sexual dysfunction (78, 98–102). On the other hand, patients with depression have manifested abnormalities of numerous hormones, including thyroid hormones, insulin, and sex hormones. This suggests that endocrine disorders may be involved in the pathology of depression (103).

Although currently, there are few studies about the effect of GD on depression from the perspective of endocrine disorders, evidence has found that hormonal abnormalities are closely related to the clinical manifestations of depression. Therefore, we hypothesized that several hormonal abnormalities in GD could increase the risk of depression, and we analyzed the specific mechanism regarding hormonal abnormality of the HPT axis, insulin metabolism, and sex hormones.

#### 3.2.1 Hormonal abnormalities in the HPT axis

The hypothalamus-pituitary-thyroid axis (HPT) is a hormone axis that regulates thyroid function by grading and feedback regulation of thyrotropin-releasing hormone (TRH), thyrotropin (TSH), thyroxine (T4), and triiodothyronine (T3). It interacts with several other hypothalamic-pituitary-target axis systems and plays a key role in maintaining homeostasis (104). Autoantibodies produced in GD can activate the TSHR pathologically and overcomes the physiological HPT negative feedback loop, leading to elevated thyroid hormones despite low levels of serum TSH (105). As the HPT axis has long been involved in the research of depression (106), it is reasonable to believe that the dysfunction of the HPT axis in GD, with its effect of disrupting neurotransmitter metabolism, inducing oxidative stress, contributing to other complications and so forth, has a potentially vital effect on the development of depression.

On the one hand, the increase in thyroid hormones can affect the metabolism of neurotransmitters in the brain. In the physiological state, thyroid hormones regulate the expression of genes needed for nerve cell differentiation, migration, and myelin formation. This plays a vital role in nerve development and signal transmission. Nevertheless, with the excessive production of thyroid hormones in GD, changes in the levels of several neurotransmitters, particularly NE and DA, will disrupt the brain’s regular signal route and thus increase the risk of depression. A significant decrease in NE and DA in the cerebral cortex of hyperthyroidism rats has been observed in experiments (107), which can further lead to depression (108, 109). On the other hand, long-term exposure to high levels of thyroid hormones is associated with oxidative stress, which is related to depression-related neurodegenerative changes or even neuro-death in the brain (110). Neuroimaging studies have found that patients with hyperthyroidism had reduced grey matter volume in portions of the occipital lobe (lateral occipital sulcus and fusiform gyrus) in the left hemisphere, the frontal and parietal lobes (insula, paracentral, precuneus, superior frontal cingulate, superior parietal and postcentral) in the right hemisphere. It has been clearly demonstrated that altered circulating thyroid hormone is associated with various mental signs and symptoms, such as emotional disturbances (impulsiveness, irritability), cognitive deficits (impairments in memory, concentration, attention, planning, and productivity), and affective symptoms (anxiety, depression, mania), which increase the incidence of neuropsychiatric disorder like depression (111). Apart from the increased thyroid hormones, clinical studies have found that decreased level of TSH is an important risk factor for depression in the elderly (112), although its underlying mechanism remains unknown.

In addition to direct effects from the hormonal abnormality of the HPT axis, complications of HPT dysfunction can also contribute to depression. A mendelian randomization study has found that hyperthyroidism can cause hemodynamic changes, which results in cardiovascular diseases represented by atrial fibrillation (AF) (113, 114). As studies have uncovered AF as a risk factor for depression (114, 115), the development of AF in the context of HPT dysfunction may increase the risk of depression. Additionally, hyperthyroidism can also cause insulin resistance (IR) and diabetes. Microvascular dysfunction is widespread in people with diabetes, which can affect the brain. A growing body of evidence suggests that microvascular dysfunction is one of the key underlying mechanisms of an increased risk of developing certain brain or mental disorders in diabetes. The microvasculature is involved in the regulation of many cerebral processes. When it is impaired, patients are more susceptible to lacunar and hemorrhagic stroke, cognitive dysfunction, and depression (116). A population-based cohort study demonstrated that vascular damage caused by microvascular dysfunction in frontal and subcortical brain regions, which are involved in mood regulation, might lead to depression in older individuals (117). Besides, cerebral microvascular dysfunction can increase BBB permeability (116). Data from human and animal studies provide strong evidence of neurovascular unit dysfunction with BBB hyperpermeability in association with oxidative stress and neuroinflammation (118), which can increase the susceptibility to neurological disorders such as depression.

In summary, elevated thyroid hormones in GD hyperthyroidism can directly increase the risk of depression. It is also noteworthy that complications of HPT dysfunction in GD can also increase susceptibility to depression.

#### 3.2.2 Insulin metabolism abnormalities

Insulin is a protein hormone secreted by islet β cells in the pancreas, stimulated by endogenous or exogenous substances such as glucose, lactose, glucagon, etc. It is the only hormone that can lower blood glucose levels. Although specific mechanisms remain unclear, thyroid hormone has the function of regulating blood glucose level in the body (119, 120). Previous studies have shown that excessive thyroid hormones can lead to IR and abnormal glucose metabolism (121).

IR refers to the inability of insulin to effectively promote glucose uptake in peripheral tissues or to inhibit hepatic glucose output, which leads to increased blood glucose. High intracellular glucose concentration develops when hyperthyroidism induces IR in GD patients, because brain cells like cerebral microvascular endothelial cells, pericytes, and astrocytes are unable to slow down their glucose transport rate (122, 123). It can then cause dysfunction of these brain cells, including increasing microvascular endothelial cells’ permeability, leucocyte adhesion, procoagulant activity, and reducing the availability of nitric oxide through various biochemical pathways initiated by mitochondrial overproduction of reactive oxygen species (124). Certain regions of the brain can become dysfunctional as a result of malfunctioning cells, which raises the risk of mental diseases. According to the vascular depression hypothesis, vascular damage in the frontal and subcortical brain regions, which are involved in mood regulation, might lead to depression in older individuals (117). On the other hand, chronic hyperglycemia due to IR leads to increased extracellular and intracellular formation of advanced glycation end products (AGEs), leading to detrimental effects, including increased oxidative stress and production of inflammatory cytokines in these brain cells (125). Oxidative stress and inflammatory cytokines can directly disrupt the BBB and damage neuronal tissue, which is likely to cause cognitive dysfunction and increase the risk of depression (116).

Furthermore, insulin has the function of enhancing memory and mediating signal transduction in plasticity and stress responses (126). Decreased insulin receptor sensitivity plays an important role in the mechanism of neuropsychiatric diseases, including depression. Insulin sensitivity in patients with hyperthyroidism has been reported to be reduced (127). The loss of insulin sensitivity of hippocampal CA1 pyramidal neurons can subsequently cause long-term Ca ^2+^-dependent afterhyperpolarizations (AHPs), which can produce significant cognitive and memory impairment in the short term and throughout life, contributing to depression (122, 123).

#### 3.2.3 Sex hormones abnormalities

Many research has discovered sex differences in the prevalence of both GD and depression, with a reported male-to-female ratio of 1:4-6 in GD and a ratio of roughly 1:2 in major depressive disorder (122, 128, 129). Moreover, women are more susceptible to both of these diseases during pregnancy and the perinatal period (130). As sex hormone levels differ greatly between significantly genders and during different physiological events of women, the evidence has pointed to the essential role that sex hormones play in both diseases.

Both estrogen(E2) and androgen can increase the risk of GD through different mechanisms. B cell activating factor (BAFF) is a B cell survival factor that promotes autoreactive B lymphocytes and inhibits their deletion (131). BAFF expression is closely linked with autoimmunity, and upregulated BAFF activity was found to contribute to the occurrence of multiple AIDs, including GD. E2 might be involved in modulating BAFF levels, resulting in a higher incidence of AITD in women (132–134). Cheng et al. (135) compared the serum BAFF levels between genders in clinical samples and discovered higher BAFF levels in women in both GD and control groups. Additionally, in the experiment of SAT mice, exogenous E2 treatment increased serum BAFF levels in male SAT mice, and higher thyroid BAFF transcripts levels were found in female SAT mice regardless of E2 treatment. Thus, abnormal estrogen metabolism, which results in increased E2 levels, may contribute to a higher occurrence of GD. On the other hand, evidence shows that androgen can inhibit the production of antigen-presenting cells in the thyroid gland, as well as the uptake and transport of autoantibodies, thereby preventing the occurrence and progression of autoimmune thyroid diseases (136). Thus, androgen depletion would increase the risk of the thyroid being subjected to an autoimmune attack, leading to an increased susceptibility to GD.

Similar effects of sex hormones were found in patients with depression, which may be related to synaptic efficacy (137). As the biological basis of learning and memory, synaptic efficacy is regulated by sex hormones. Female estrogen changes periodically, affecting the structure and function of the brain, resulting in female-unique brain plastic changes and related vulnerability (130). As one of the most basic and essential functions of the brain, synaptic plasticity affects the ability to perceive, evaluate and store complex information and guides the body to make appropriate adaptive responses to subsequent stimuli. It plays a crucial role in short-term and long-term memory. Many factors, such as the interruption of the estradiol cycle and the increase of inflammatory cytokines, will lead to changes in synaptic function and morphology, which may be one of the pathogenesis of depression (138).

It is noteworthy that when sex hormone disorders cause GD, it may also contribute to depression, resulting in the comorbidity of both diseases. Furthermore, sex hormone disorders during the onset of GD are likely to affect the normal metabolism of sex hormones, which might lead to or aggravate the depressive state of GD patients. Further research into this field will help provide deeper insight into the interaction between the two diseases.

### 3.3 A possible thyroid-gut-microbiome-brain axis increases the risk of depression

The human microbiota has been found to be a key regulator of health and diseases, and more and more evidence has pointed out their relevance to many human diseases (139). As the primary interface, the gastrointestinal tract contains about 66% of the human microbiota (139), including bacteria, viruses, fungi, and so on (140, 141). The gut microbiota has been implicated in numerous human diseases (142). The bidirectional pathways between the gut and the brain, known as the gut-microbiome-brain axis, have long been recognized. Research has shown a strong correlation between the gut microbiota and the pathophysiology of depression and anxiety (143–146). In recent years, there has been increasing awareness of the role of microbiota in the pathogenesis of GD (139, 147–149). Current evidence has pointed to shared microbiota changes in GD and depression, indicating that gut microbiome dysbiosis in GD may act as a trigger for depression (26, 150–154). A prospective clinical study with 39 participants with GD and 17 without GD found that the levels of bacilli, Lactobacillales, Prevotella, Megamonas (154). Hou et al. (155) summarize the studies of 263 Graves Disease(GD)/239 Health Control(HC) samples until July 2021. In general, about 29 gut microbiota taxa are different in GDs compared with HCs. Though results of some microbiota taxa are not consistent in different studies, several taxa, including Prevotellaceae and Veillonellaceae at the family level and Bacteroides, Lactobacillus, Prevotella, and Veillonella at the genus level were identified in three or more studies. Among these studies, it is noteworthy that Prevotellaceae and Prevotella were identified as the core microbiome of the GD group and were closely associated with GD patients. On the other hand, the microbiota alterations in depression include the decline of both the microbiota diversity and richness (150, 156). Interestingly, research have revealed that the abundance of Prevotellaceae and Prevotella increased in depressed patients, indicating that gut microbiome dysbiosis in GD is likely to contribute to the microbiota alteration in depression. Given the above correlation, we predict that a possible thyroid-gut-microbiome-brain axis is involved in the pathogenesis of mental disorders, thereby increasing the risk of depression.

#### 3.3.1 Gut microbiome dysbiosis in GD induces unbalanced neurotransmitters in the brain

Neurotransmitters like serotonin [also known as 5-hydroxytryptamine (5-HT), dopamine (DA), and noradrenaline (NE)] have been implicated in the pathophysiology of depression for many years as essential components of the brain’s function. Under normal conditions, the gut microbiota secretes neurotransmitters (e.g., Gamma-Amino Butyric Acid - GABA, serotonin) and trophic factors (e.g., brain-derived neurotrophic factor - BDNF), which is related to neuronal communication, maintenance, and survival of the brain through neurotrophic support. Although the exact mechanism of how gut microbiota influences neurotransmitters in depression is still unclear, some hypotheses and research have pointed out that neurotransmitter imbalance might play a vital role in the process. In the monoaminergic neurotransmitter deficiency hypothesis, it is posited that depressive symptoms arise from insufficient levels of monoamine neurotransmitters such as 5-HT, norepinephrine (NE), and/or dopamine (DA) (157). Subsequent research has added that the hyperactivity of glutamatergic system (158) and acetylcholine system (159), the inhibition of the gamma-aminobutyric acid (GABA) system (160) also contribute to depression.

Among the neurotransmitters related to depression, GABA is the main inhibitory mediator in the CNS, which is crucial in controlling various psychological and physiological processes. These processes include antianxiety, diuresis, and neurotransmission (161). Multiple neurological and psychiatric illnesses, including epilepsy, depression, and Alzheimer’s disease, are linked to the etiology of abnormalities in GABA levels and GABA receptor functioning in the central nervous system (162). Researchers have discovered that the GABA system is inhibited in the pathogenesis of depression (51, 163, 164), resulting in less secretion of GABA in the brain. The gut microbiome is an important regulator of GABA synthesis, with Lactobacillus brevis being the most effective enteral flora generator of GABA (165).

Research has reported the reduction of Lactobacillus in the onset of depression, resulting in less production of GABA, which is likely to contribute to depression (166). On the other hand, three studies have reported the increase of Lactobacillus in GD patients and the positive correlation between TRAb level and Lactobacillus. While Lactobacillus is often not harmful in the human body and may exhibit an antidepressant effect at the start of GD, some research has revealed that Lactobacillus might cause macrophages to release pro-inflammation cytokines including IL-6 and TNF-α (167). Thus, according to the cytokine hypothesis, it is reasonable to believe that these pro-inflammatory cytokines can then increase the risk of depression through ways such as inhibiting the negative feedback of the HPA axis, increasing the permeability of the BBB, and so on. Whether the increase of Lactobacillus in GD plays a protective or pathogenic role in the pathogenesis of depression remains to be further studied.

Other neurotransmitters related to gut microbiota may also contribute to depression in the onset of GD. Serotonin, or 5-HT, is a tryptophan (Trp) metabolite that plays a significant role in GI (gastrointestinal) regulation and has a variety of physiological effects on both humans and animals (168). Serotonin exists and is stored in two distinct pools in the human body, namely the peripheral and the central pool. Unlike peripheral 5-HT, central 5-HT performs critical control functions in the brain. It is a key regulator of behaviors such as emotion, stress response, appetite, addiction, and pain (169). It is also an important participant in the modulation of neuronal differentiation and migration, axonal outgrowth, myelination, and synapse formation (170). Decreased availability of 5-HT in the brain is a key characteristic of depression (170). Since peripheral 5-HT cannot cross the BBB, central serotonin is synthesized from Trp, which is transported through the BBB from blood circulation (170). As a crucial role in supplying our body with the required amount of Trp, the gut microbiota can regulate the CNS by modulating the central 5-HT level.

Research has revealed several pathways in which gut microbiota alterations in GD may contribute to a decreased level of central serotonin, thereby increasing the susceptibility to depression. On the one hand, reduced short-chain fatty acids (SCFAs) in GD patients may contribute to the reduction of central serotonin concentration. According to a research on gut microbiota in GD patients by gas chromatography-mass spectrometry (GC-MS), propionic acid and butyric acid, two prominent SCFAs, were considerably lower in GD patients (153). SCFA-producing Bacteroides fragilis YCH46 strain (B.f.S) has also been discovered to be significantly reduced in GD patients (153). SCFAs, particularly butyrate, which can be transported into blood circulation, are reported to increase the brain serotonin concentration, providing neuroprotective benefits to stressed mice (171). Consequently, reduced level of SCFAs in GD patients is likely to result in decreased brain serotonin concentration, which increases the risk of depression. On the other hand, tryptophan and serotonin metabolism alterations in GD patients are also likely to lower the central 5-HT level. Several recent studies have pointed out that alterations of the gut microbial tryptophan metabolism influence peripheral Trp availability, affecting central tryptophan levels and, thereby, leading to changes in the central serotonin metabolism (171, 172). Some other research discovered that enhanced serotonin metabolism may also contribute to decreased central serotonin levels following gut dysbiosis (173). It should be noted that the exact mechanism of tryptophan and serotonin metabolism alterations in GD still needs to be clarified. Thus, further studies into this aspect will establish a more profound correlation between GD and depression induced by 5-HT reduction.

#### 3.3.2 Gut microbiome dysbiosis in GD activates host immune response

The gut microbiota plays a vital role in the formation and function of the host’s immune system. As mentioned in the previous sections, autoimmune responses play a critical role in the development of depression in GD. However, it is not yet clear how autoimmune responses of GD are activated. Given the gut microbiota’s critical role in the immune system, gut dysbiosis can induce immune responses and contribute to the autoimmune responses of GD, which is correlated with an increased risk of depression.

The main immune response is the imbalance between Th17 and regulatory T cells (Tregs). Under healthy conditions, gut microbiota can maintain the balance between Th17/Tregs. But in GD patients, dysbiosis may increase Th17 cells and suppress Treg production, manifested by the decrease of CD4^+^Foxp3^+^ Treg cells and the increase of CD4^+^IL-17^+^ Th17 cells in circulation (153, 174). Additionally, Bacteroides fragilis YCH46 strain (B.f.S), which can increase Tregs, IL-10, reduce Th17 cells and IL-17A levels in peripheral blood mononuclear cells(PBMCs) from healthy individuals, significantly decreased in GD patients, resulting in decreased IL-10 and elevated IL-17A levels (153).

Although the role of Th17 in depression has not been fully clarified, and research on the correlation between IL-17 and depression is still limited, accumulating evidence have pointed to the involvement of Th17 and IL-17 in depression. Chen et al. have observed increased circulating Th17 cells in depressed patients (175). The exact influence of circulating Th17 cells on depression remains unclear, but since Th17 cells can infiltrate the brain parenchyma without requiring VLA4 signals, it can be hypothesized that Th17 cells may reach the brain and synthesize IL-17 (68). On the other hand, two studies have found that circulating IL-17A is elevated in depressed patients (175, 176), and the administration of IL-17A is able to promote depressive-like behaviors (177). As evidence has shown that IL-17 can increase depression and depression-like behaviors through the activation of microglia and inducing neuronal death, we can speculate that increased Th17 cells and IL-17A, induced by gut dysbiosis during the onset of GD are involved in the immune response of depression, exacerbating depressive symptoms. More complex immune interactions between gut dysbiosis in GD and depression will require further investigations in this field.

## 4 Therapeutic approaches for decreasing depression risk among individuals with GD

Reports have covered that GD is often complicated with psychiatric symptoms and diseases, such as depression, anxiety, and schizophrenia (178, 179). These neuropsychiatric symptoms and illnesses not only seriously impair the quality of life of patients with GD, but also aggravate the original condition of GD and affect its prognosis (180, 181).

In view of this, more attention should be paid to the mental and psychological status of GD patients, and preventive measures should be taken to avoid the occurrence of depression during GD. Although current guidelines do not specifically address the management of depression in GD patients, several approaches can be adopted in light of the current understanding of both conditions. Close monitoring and routine evaluations of the patient’s mental state are necessary to prevent the detrimental effects of GD on mental health. Previous studies have applied the standard Mini-International Neuropsychiatric Interview or the Hamilton Depression Rating Scale and the Beck Depression Inventory for evaluating the mood state of GD patients, which can be potential methods for assessing the patients' mental state (182).

In terms of treatment options, it is notable that early diagnosis and treatment, as well as GD progression management using standard methods recommended by guidelines might lower the occurrence of mental disorders. Besides, previous research has pointed out differences in gut microbiome in patients with depression and indicated the potential treatment method with probiotics and symbiotics (183). Given the shared microbiota changes in GD and depression (26, 150–154), as well as the potential effects of microbiota dysbiosis in GD on depression, probiotics and symbiotics may exert beneficial effects in improving the intestinal flora environment of patients with GD, thus reducing the risk of depression. Additionally, another research reveals that methimazole (MMI) combined with probiotics improves the level of TRAb, which will play a positive role in lowering the recurrence rate of GD and is better suited to sustaining the positive emotion of GD patients (184). Since they are rarely implied in the current clinical treatment of GD, we recommend that probiotics and symbiotics should be utilized to improve depressive disorders in GD patients. Medicines used in the treatment of hyperthyroidism have also been shown to have potential beneficial effects in relieving depressive symptoms. Clinical practice suggests that antithyroid drugs combined with β-adrenergic receptor antagonists can be used as effective drugs for the treatment of mental disorders and psychiatric symptoms caused by hyperthyroidism, as the excessive activation of the adrenal system induced by hyperthyroidism can lead to somatic symptoms (14).

Additionally, psychosomatic synchronization therapy is considered to be suitable for GD patients with psychiatric symptoms (185), as research has shown that the combination of antithyroid, antipsychotic drugs and psychotherapy contribute to the prognosis of the disease (186). Also, psychological treatment can be supplemented when patients exhibit mental symptoms such as depression. However, due to the complexity of their pathophysiological mechanisms, treatments and interventions for depressive symptoms in GD patients is still under development, and further research is needed to assess the efficacy of these therapies (187).

## 5 Conclusions

GD, as the most common cause of hyperthyroidism, is often manifested as emotional agitation and irritability. In recent years, increasing evidence has pointed out the high occurrence of depression in GD, but it has not received adequate attention. Although currently only a few clinical studies have been conducted on the relationship between GD and depression, evidence has been able to point out the strong correlation between GD and depression. The pathophysiological mechanisms, including inducing autoimmune responses, hormonal disorders, and exerting an effect on the possible thyroid-gut-microbiome-brain axis, have underpinned the role of GD as a crucial driver in the development of depression and depressive symptoms. This can illuminate better management and treatment methods in terms of avoiding depression in GD patients and treating patients with depressive complications. We hope that through reviewing the potential mechanism of depression in GD, more attention can be drawn to the prevention and treatment of psychiatric complications of GD, so as to improve the treatment and prognosis of the disease.

## Author contributions

YS and XW wrote the manuscript. WM, JS, XZ, YT, and YY contributed to the provided guidance of the whole manuscript. WM, XZ and YT reviewed and revised the manuscript. JS, YS, and SY prepared the figures. All authors contributed to the article and approved the submitted version.
